# Biophysical Investigation of the Membrane-Disrupting Mechanism of the Antimicrobial and Amyloid-Like Peptide Dermaseptin S9

**DOI:** 10.1371/journal.pone.0075528

**Published:** 2013-10-11

**Authors:** Lucie Caillon, J. Antoinette Killian, Olivier Lequin, Lucie Khemtémourian

**Affiliations:** 1 UPMC Univ Paris 06, UMR 7203 CNRS-UPMC-ENS, Laboratoire des Biomolécules, Paris, France; 2 Research Group Membrane Biochemistry & Biophysics, Bijvoet Center and Institute of Biomembranes, Utrecht University, Utrecht, The Netherlands; University of Cambridge, United Kingdom

## Abstract

Dermaseptin S9 (Drs S9) is an atypical cationic antimicrobial peptide with a long hydrophobic core and with a propensity to form amyloid-like fibrils. Here we investigated its membrane interaction using a variety of biophysical techniques. Rather surprisingly, we found that Drs S9 induces efficient permeabilisation in zwitterionic phosphatidylcholine (PC) vesicles, but not in anionic phosphatidylglycerol (PG) vesicles. We also found that the peptide inserts more efficiently in PC than in PG monolayers. Therefore, electrostatic interactions between the cationic Drs S9 and anionic membranes cannot explain the selectivity of the peptide towards bacterial membranes. CD spectroscopy, electron microscopy and ThT fluorescence experiments showed that the peptide adopts slightly more β-sheet and has a higher tendency to form amyloid-like fibrils in the presence of PC membranes as compared to PG membranes. Thus, induction of leakage may be related to peptide aggregation. The use of a pre-incorporation protocol to reduce peptide/peptide interactions characteristic of aggregates in solution resulted in more α-helix formation and a more pronounced effect on the cooperativity of the gel-fluid lipid phase transition in all lipid systems tested. Calorimetric data together with ^2^H- and ^31^P-NMR experiments indicated that the peptide has a significant impact on the dynamic organization of lipid bilayers, albeit slightly less for zwitterionic than for anionic membranes. Taken together, our data suggest that in particular in membranes of zwitterionic lipids the peptide binds in an aggregated state resulting in membrane leakage. We propose that also the antimicrobial activity of Drs S9 may be a result of binding of the peptide in an aggregated state, but that specific binding and aggregation to bacterial membranes is regulated not by anionic lipids but by as yet unknown factors.

## Introduction

The increasing resistance of pathogens against conventional antibiotics has become a major concern for treatment of infectious diseases. Therefore, development of new classes of therapeutic agents is urgently required. One group of molecules that has attracted interest are cationic antimicrobial peptides. Unlike conventional antibiotics that bind to a target in the membrane or cytosol and act specifically on biochemical pathways, most antimicrobial peptides kill cells by permeabilizing the membrane through peptide-lipid interactions. Many studies have been conducted with antimicrobial peptides, and various membrane-destabilization mechanisms were proposed such as the “barrel-stave” and the “carpet” models. The former postulates that the peptide acts by perturbing the barrier function of membranes through transmembrane pore formation [1]. In the “carpet” model, the peptide acts in a detergent-like manner, covering the membrane surface until a threshold concentration is reached leading to membrane pore formation and finally to membrane disruption [2]. Other specific mechanisms have also been suggested, including phase separation due to specific peptide-lipid interactions [3], [4] and detergent-like solubilization of the membrane [5]. All these mechanisms are not mutually exclusive and an individual peptide may induce different mechanisms of membrane damage depending on the membrane lipid composition [6], [7].

Frog skin is by far the most important source of antimicrobial peptides, with more than half of the peptides described to date isolated from South American Hylidae and European, Asian or North American Ranidae [8]. Among these peptides, dermaseptins B and S, secreted in the skin of the South American tree frogs *Phyllomedusa bicolor* and *P. sauvagei* respectively, form a family of amphipathic, α-helical, closely related antimicrobial peptides with broad-spectrum microbicidal activities at micromolar concentrations against Gram-positive and Gram-negative bacteria, fungi, yeasts and protozoa [9]. One of these peptides, dermaseptin S9 (Drs S9), a cationic peptide, does not resemble any antimicrobial peptide identified to date, but is more similar to synthetic peptides that were originally designed as transmembrane mimetic model peptides [10]–[12], having a hydrophobic core sequence flanked at both termini by several positively charged residues. Usually, cationic antimicrobial peptides are unstructured in buffer, but fold into α-helical structure in the presence of lipids. Consistent with this, previous structural studies showed that Drs S9 folds into an α-helical structure in TFE/water mixtures, while the NMR spectrum in water shows very broad signals indicative of aggregation in aqueous solution [13]. Subsequently, it was shown that Drs S9 slowly assembles into amyloid-like fibrils at 500 µM in aqueous solution and that the antimicrobial and chemotactic activities of Drs S9 are modulated by its propensity to form fibrils [14]. Indeed, Drs S9 exhibited an antimicrobial activity against all Gram-negative bacteria strains tested at day 0 and day 3 whereas the peptide exhibited little or no antibacterial activity after 7 days of incubation. Surface plasmon resonance experiments indicated that Drs S9 binds to anionic or zwitterionic phospholipid vesicles [13]. This Drs S9/lipid interaction may be important for the antimicrobial activity of the peptide, for example because membranes may modulate the conformation or the fibril forming propensity of the peptide, and/or because the peptide may interfere with the permeability properties of the membrane. Despite this potential biological importance, to our knowledge these two studies [13], [14] are the only ones so far on the interaction between membranes and Drs S9.

To gain further insight into the nature of the initial steps of Drs S9-membrane interactions, including the potential importance of lipid composition, we performed a biophysical study of Drs S9 in lipid bilayers of different compositions. In particular, we used model membranes of the zwitterionic lipid phosphatidylcholine (PC) and the anionic lipid phosphatidylglycerol (PG) in a 7∶3 ratio to mimic the lipid composition of microorganism plasma membranes and we used bilayers composed of pure PC and PG to analyze the importance of electrostatic interactions. To further understand the importance of amyloid-like fibril formation in relation to membrane-structure and membrane-damage induced by Drs S9, we also studied and compared the effect of addition of the peptide to preformed membrane vesicles versus intimate incorporation into the membranes via cosolubilization of peptide and lipid in organic solvent.

## Experimental Procedures

### Materials

Dermaseptin S9 (GLRSKIWLWVLLMIWQESNKFKKM) was synthesized using Fmoc chemistry at the Institut de Biologie Intégrative (IFR83) at the University Pierre et Marie Curie. The peptide was purified by reverse phase high-performance liquid chromatography (HPLC). The purity of the peptide was higher than 95%, as determined by analytical HPLC and the mass of the peptide was confirmed with MALDI-TOF mass spectrometry. 1,2-dioleoyl-*sn*-glycero-3-phosphocholine (DOPC), 1,2-dimyristoyl-*sn*-glycero-3-phosphocholine (DMPC) and 1,2-dimyristoyl-*sn*-glycero-3-phospho-1′-*rac*-glycerol (DMPG) (obtained from Genzyme, Switzerland) and 1,2-dioleoyl-*sn*-glycero-3-phospho-1′-*rac*-glycerol (DOPG), 1-palmitoyl-2-oleoyl-*sn*-glycero-3-phospho-1′-*rac*-glycerol (POPG) and palmitoyl chain deuterated 1-palmitoyl-2-oleoyl-*sn*-glycero-3-phosphocholine (POPC-d_31_) and 1-palmitoyl-2-oleoyl-*sn*-glycero-3-phospho-1′-*rac*-glycerol (POPG-d_31_) (obtained from Avanti Polar Lipids, Alabaster, USA) were used without further purification. Calcein was obtained from Sigma.

### Preparation of MLVs and LUVs

Lipid films were made by dissolving the desired lipids in chloroform (for DOPC, POPC and DMPC) or chloroform/methanol 3∶1 (for DOPG, POPG and DMPG). The solvent was evaporated under dry nitrogen gas. The resulting films were then kept under vacuum desiccator for at least 30 minutes. Films were then rehydrated with appropriate buffer (10 mM Tris-HCl, 100 mM NaCl, pH 7.4 for the calcein leakage assay, DSC and NMR experiments and 10 mM sodium phosphate buffer, pH 7.4 for the CD experiments) at a temperature above the transition temperature of the lipids for 30 minutes. The lipid suspensions were frozen in liquid nitrogen and thawed in a 40°C water bath 3 times to obtain homogeneous multilamellar vesicles (MLVs). Large unilamellar vesicles (LUVs) with a mean diameter of 200 nm were prepared from the MLVs using the following protocol: the lipid suspensions were subjected to 10 freeze-thaw cycles, and passed 19 times through a mini-extruder (Avanti Polar Lipids, Alabaster, USA) equipped with a 200 nm polycarbonate membrane. The phospholipid content of lipid stock solutions and vesicle preparations was determined as inorganic phosphate according to Rouser [15]. Calcein-containing LUVs were made using the same protocol, except for the following adaptations. The buffer for hydration of the lipid films was replaced by a solution containing 70 mM calcein and 10 mM Tris-HCl (pH 7.4). Free calcein was separated from the calcein-filled LUVs using size-exclusion chromatography (Sephadex G50-fine) and elution with 10 mM Tris-HCl, 100 mM NaCl (pH 7.4).

### Peptide preparation

When Drs S9 was incorporated into the vesicles, lipid and freshly peptide were solubilized in respectively CHCl_3_/CH_3_OH and TFE and then mixed together. The organic solvents were removed under high vacuum and the residual lipid/peptide film was hydrated in the appropriate buffer (pH 7.4). For experiments where Drs S9 was added to lipid vesicles, the preformed vesicles were slurried with a 1 mM peptide solution in milliQ water. A peptide:lipid molar ratio of 1∶20 was used in all experiments.

### Thioflavin assays

The kinetics of Drs S9 fibril formation were measured using the fluorescence intensity increase upon binding of the fluorescent dye Thioflavin T (ThT) to fibrils. A plate reader (Fluostar Optima, Bmg Labtech) and standard 96-well flat-bottom black microtiter plates in combination with a 440 nm excitation filter and a 485 nm emission filter were used. The ThT assay was started by adding 10 µL of a 2 mM Drs S9 (100 µM peptide) in aqueous solution to 190 µL of a mixture of 10 µM ThT, LUVs (2 mM lipids; peptide:lipid ratio 1∶10) and 10 mM Tris/HCl, 100 mM NaCl (pH 7.4). The microtiter plate was shaken for 10 seconds directly after addition of all components, but not during the measurement. The ThT assay was performed 2 times, each in duplicate. The results presented here are the average of the different experiments; errors bars indicated the statistical dispersions.

### Electron microscopy

Peptides and LUVs were incubated under the same conditions as in the Thioflavin T assay. Aliquots (25 µL) of this mixture were adsorbed onto glow-discharged carbon-coated 300-mesh copper grids for 2 min. Grids were then blotted and dried. Grids were negatively stained for 45 s on 2% uranyl acetate, blotted and dried. Grids were examined using a Jeol 2100 electron microscope operating at 200 kV.

### Calcein permeability assay in large unilamellar vesicles

A plate reader (Fluostar Optima, Bmg Labtech) was used to perform calcein leakage experiments in standard 96-well transparent microtiter plates. Measurements were conducted on calcein-loaded DOPC, DOPC/DOPG 7∶3 and DOPG LUVs. The Drs S9 was added to a mixture of calcein-containing LUVs in 10 mM Tris-HCl, 100 mM NaCl, pH 7.4 buffer. The final concentrations were 20 µM for lipids and 1 µM for peptide (peptide:lipid ratio of 1∶20). Directly after addition of all components, the microtiter plate was shaken for 10 s using the shaking function of the plate reader. The plate was not shaken during the measurement. Fluorescence was measured from the bottom, every minute, using a 485 nm excitation filter and a 520 nm emission filter. The temperature was approximately 28 °C ± 3 °C. The maximum leakage at the end of each measurement was determined via addition of 1 µL of 10% Triton-X100 to a final concentration of 0.05% (v/v). The release of fluorescent dye was normalized according to the following equation: 
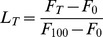



In this equation, L_T_ is the fraction of dye released (normalized membrane leakage), F_T_ is the measured fluorescence intensity, and F_0_ and F_100_ are the fluorescence intensities at time zero and after addition of Triton-X100, respectively. The calcein leakage experiment was performed 4 times, each in triplicate, on different days. The results presented here are the average of the different experiments, ± standard deviation.

### Monolayer experiments

Peptide-induced changes in the surface pressure of a monomolecular layer of phospholipids at constant surface area were measured with the Wilhelmy plate method, as reported previously [16], [17]. Surface pressures were measured at room temperature. Briefly, a trough was filled with 5.5 mL of 10 mM Tris–HCl, 100 mM NaCl buffer (pH 7.4). DOPC, DOPC/DOPG (7∶3) and DOPG monolayers were spread from a 0.5 mM stock solution in chloroform or chloroform/methanol (3∶1). The lipid monolayer was allowed to stabilize for a few minutes before 5 µL of 1.1 mM freshly prepared stock solution of the peptide was injected into the sub–phase without disturbing the lipid monolayer. The final peptide concentration was 1 µM.

### Circular dichroism

CD spectra were recorded on a Jasco 815 spectropolarimeter (Jasco Inc., Easton, MD) over the wavelength range 190–270 nm, at 0.2 nm intervals and 20 nm.min^−1^ scan speed. Five scans were accumulated and averaged. Temperature was kept at 25°C. Spectral measurements were performed in 1 mm path length quartz cells from Hellma GmbH, using DOPC, DOPC/DOPG 7∶3 and DOPG LUVs, with added or incorporated peptide. Peptide concentrations were 25 µM in the absence and in the presence of lipids (peptide:lipid ratio 1∶20). CD measurements are reported as molar ellipticity per residue (degree.dmol^−1^.cm^2^.residue^−1^), and are given by:
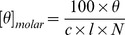
where θ is the recorded ellipticity in degrees, c is the peptide concentration in dmol.L^−1^, l is the cell path-length in cm and N is the number of residues. In order to estimate the peptide secondary structure content, an analysis of relevant CD spectra was carried out using the CDFriend program, developed by Dufourc and Buchoux [18] and CDPro software, developed by Sreerama and Woody [19]. Similar results are obtained for both methods. Results are given using CDFriend, which uses standard curves for α-helix, β-sheet and random coil obtained from L*i*K*j* peptides of known length, secondary structure and CD spectrum and therefore is more relevant for our study [20], [21]. The program implements a simulated annealing algorithm to obtain the best combination of α-helix, β-sheet, and random coil conformation that exhibits the lowest normalized root mean-square deviation (nrmsd) with respect to the experimental spectrum. The experimental error is estimated to be ± 5%.

### Differential scanning calorimetry

DSC experiments were performed on a high-sensitivity calorimeter (TA instruments, Guyancourt, France). The temperature range was 0–40°C with a scan rate of 1°C/min and a delay of 10 min between sequential scans in a series to allow thermal equilibration. Four heating and cooling scans were performed for each analysis. These experiments were performed on DMPC, DMPC/DMPG 7∶3 and DMPG MLVs, with added or incorporated peptide. The lipid concentration was 1 mg/mL with a peptide/lipid molar ratio of 1∶20. Data analysis was performed by the fitting program NanoAnalyze provided by TA instruments.

### Sample preparation for NMR experiments

Dermaseptin S9 was incorporated to MLVs of POPC-d_31_, POPC-d_31_/POPG 7∶3 or POPG-d_31_ to a final peptide:lipid ratio of 1∶20. Typically, about 10 mg of lipid was used per sample. Lipids and peptide were co-dissolved in organic solvent, evaporated under dry nitrogen gas and then kept under vacuum for at least 30 min. The residual film was suspended in 500 µL of milliQ water, and immediately lyophilized. Samples were rehydrated with 150 µL of 10 mM Tris-HCl, 100 mM NaCl buffer (pH 7.4) in deuterium-depleted water (Eurisotop, France) and then subjected to three freeze/thaw cycles. Samples were transferred into a 4 mm rotor (100 µL) for NMR experiments.

### Solid state NMR spectroscopy

NMR experiments were carried out on a Bruker Avance (Wissembourg, France) 800 MHz spectrometer operating at 122.8 MHz for ^2^H-NMR and 323.9 MHz for ^31^P-NMR. Samples were allowed to equilibrate for 30 min at 25°C before the NMR signal was acquired. Deuterium NMR experiments were performed by means of the quadrupolar echo pulse sequence 90°*x*-*τ*-90°*y-τ*-acq [22]. ^31^P-NMR spectra were acquired using a phase-cycled Hahn-echo pulse sequence with gated broadband proton decoupling [23]. Typical acquisition parameters for ^2^H-NMR experiments were as follows: spectral width of 500 kHz; π/2 pulse width of 4.5 µs; interpulse delays of 40 µs; a recycle delay of 2 s was used and 2–3 k scans were recorded. Typical acquisition parameters for ^31^P-NMR experiments were as follows: spectral width of 200 kHz, π/2 pulse width of 6.5 µs, interpulse delays of 40 µs and a recycle delay of 5 s; 2 k scans were accumulated. An exponential line broadening of 100–200 Hz was applied prior to Fourier transformation. Phosphorous chemical shifts were referenced relative to external 85% H_3_PO_4_ (0 ppm). ^2^H spectra were de-Paked according to the “de-Pake-ing” procedure implemented by Bloom *et al*. [24]. Carbon-deuterium order parameters, S_CD_, were directly extracted from quadrupolar splittings Δν_Q_ using the formula:



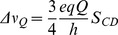
where e^2^qQ/h is the static deuterium quadrupolar coupling constant (167 kHz for C-^2^H bonds [25]). S_CD_ calculations were performed using a PYREX/PYTHON program [26].

## Results

### Drs S9 induces membrane leakage to a lesser extent in PG than in PC vesicles

**Figure 1:**
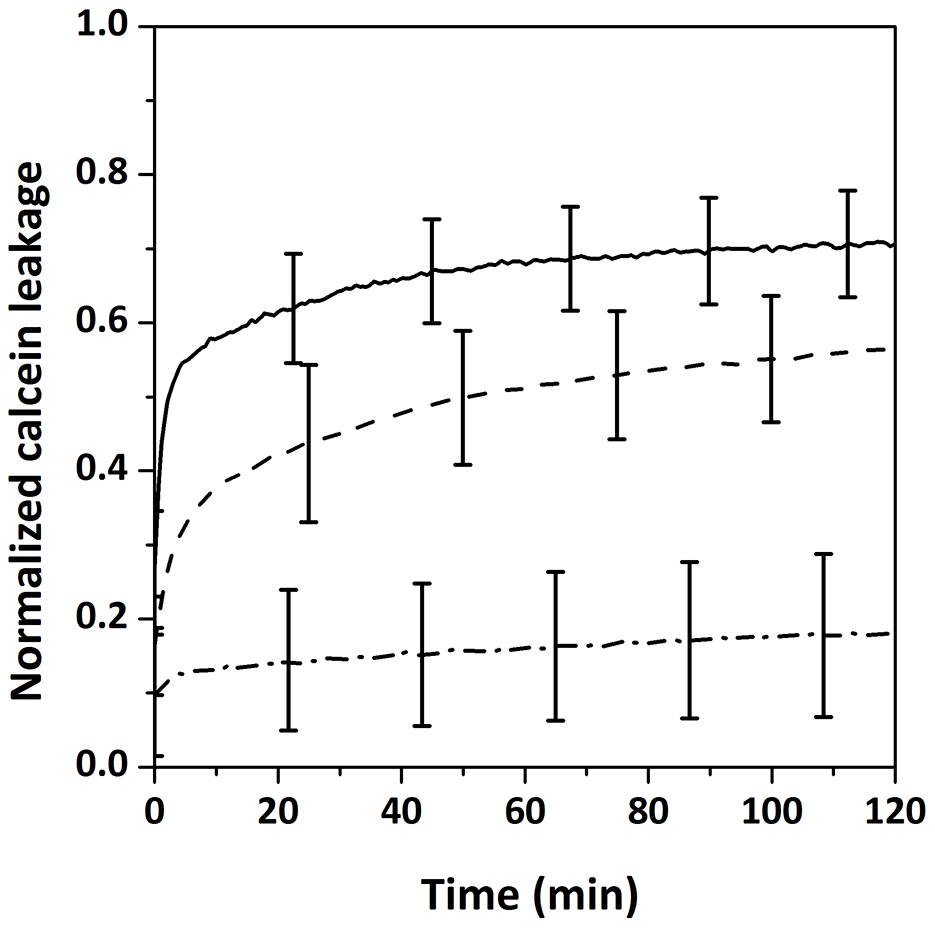
Membrane permeabilization induced by 1 µM Drs S9 of calcein-containing DOPC (solid line), DOPC/DOPG 7∶3 (dashed line) or DOPG (dashed dotted line) LUVs. Data are the average (± standard deviation) from 4 independent experiments performed in triplicate.

First the effect of the peptide was examined on membrane barrier properties in lipid vesicles of different compositions. Membrane damage was assayed quantitatively by analysing the extent of leakage of a fluorescent dye (calcein) entrapped in large unilamellar vesicles (LUVs), which is an established routinely used method to measure membrane damage [4]. The high concentration of calcein inside intact vesicles leads to self-quenching. Disruption of the membrane of the vesicle by the peptide allows calcein to escape, eliminating the self-quenching effect and therefore increasing fluorescence of calcein. Figure 1 shows that Drs S9 induces within a few minutes a significant leakage of DOPC LUVs at a concentration of 1 µM, which is of the same range as the observed minimal inhibitory concentration (MICs) [13]. The extent of leakage reaches 71 ± 7% of the total vesicle content. In DOPC/DOPG vesicles, the process of permeabilization is slightly slower than in presence of DOPC vesicles, and leads to a smaller extent of leakage (57 ± 8%). In contrast, the results obtained in the presence of DOPG LUVs are quite different; Drs S9 induces only weak membrane damage, with 18 ± 11% leakage. This result is unexpected because Drs S9 has an overall positive charge and hence would be likely to be attracted more strongly to anionic lipids. Therefore, it is likely that the observed differences in membrane leakage between the three systems (DOPC, DOPG and DOPC/DOPG) are caused by differences in membrane insertion and/or differences in conformational behavior of the peptide.

### Drs-S9 inserts less efficiently in PG than in PC monolayers

**Figure 2:**
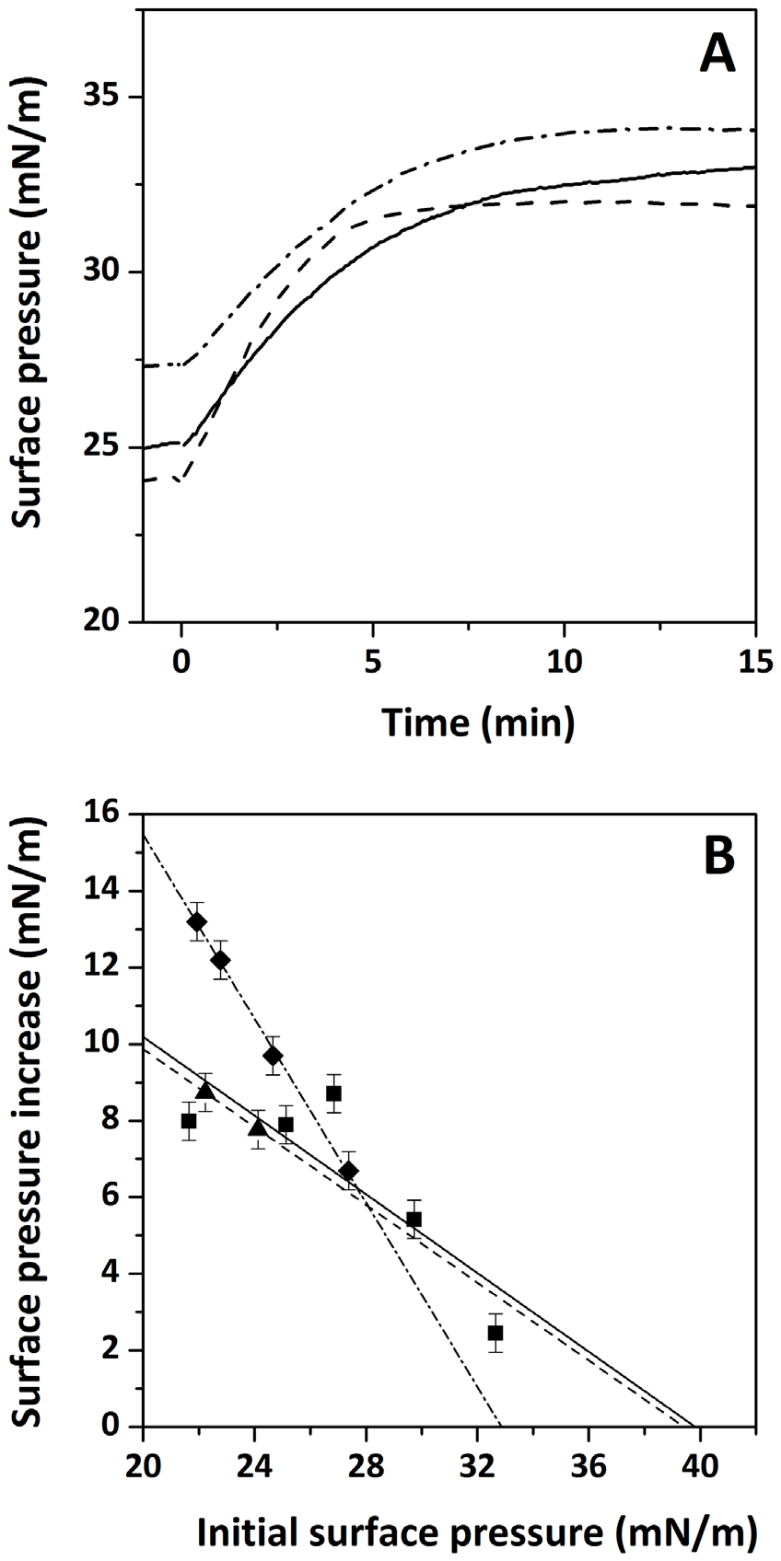
Surface pressure profile (A) after injecting a sample of Drs–S9 into a monolayer of DOPC (solid line), DOPC/DOPG (dashed line) or DOPG (dashed dotted line). The peptide was injected into the stirred sub–phase at t = 0 min. Surface pressure increase (B) induced by the interaction of Drs–S9 with DOPC (squares, solid line), DOPC/DOPG (triangles, dashed line) or DOPG (diamonds, dashed dotted line) monolayers as a function of the initial surface pressure. The straight lines were obtained by linear regression. Experimental error is estimated at ± 0.5 mN/m.

We first performed membrane surface pressure measurements on monolayers to study the insertion of the peptide in lipid membranes. Injection of Drs S9 into the aqueous sub-phase below a lipid monolayer (DOPC, DOPC/DOPG or DOPG) resulted in a fast increase in the surface pressure. After 15 min, at an initial pressure of 32–34 mN/m, the increase in surface pressure induced by addition of Drs S9 was 7.9 ± 0.5 mN/m for DOPC and DOPC/DOPG compared with an increase of 6.7 ± 0.5 mN/m for DOPG (Figure 2A), indicating an efficient insertion for Drs S9 in the three lipid systems. Then, we determined the maximal initial surface pressure at which the peptide was still able to insert, by analysing the surface pressure increase as function of the initial surface pressure. Figure 2B indicates that the extrapolated “limiting surface pressure” below which Drs S9 is able to insert is higher for DOPC and DOPC/DOPG monolayers (about 40 mN/m) than for DOPG monolayers (33 mN/m). In DOPC and DOPC/DOPG monolayers, the limiting surface pressure is significantly higher than the surface pressures that correspond to the packing density in lipids found in biological membranes, being between 31 mN/m and 35 mN/m [27], indicating that *in vivo* Drs S9 could insert efficiently into these membranes. Noteworthy, the limiting surface pressure of Drs S9 in DOPC and DOPC/DOPG is of the same order compared to that of other antimicrobial peptides [28], [29]. In contrast, in DOPG monolayer the limiting surface pressure is lower, suggesting that *in vivo* Drs S9 may not insert in pure DOPG membrane or in membrane containing PG-rich domains. These results are in agreement with calcein leakage experiments in which only a small leakage of DOPG vesicles is induced by Drs S9. The observed differences in membrane insertion between the three systems (DOPC, DOPG and DOPC/DOPG) may be caused by differences in aggregation or conformation of the peptide. Its tendency to aggregate was investigated next.

### Drs S9 form amyloid-like fibrils in solution and in the presence of membranes

**Figure 3:**
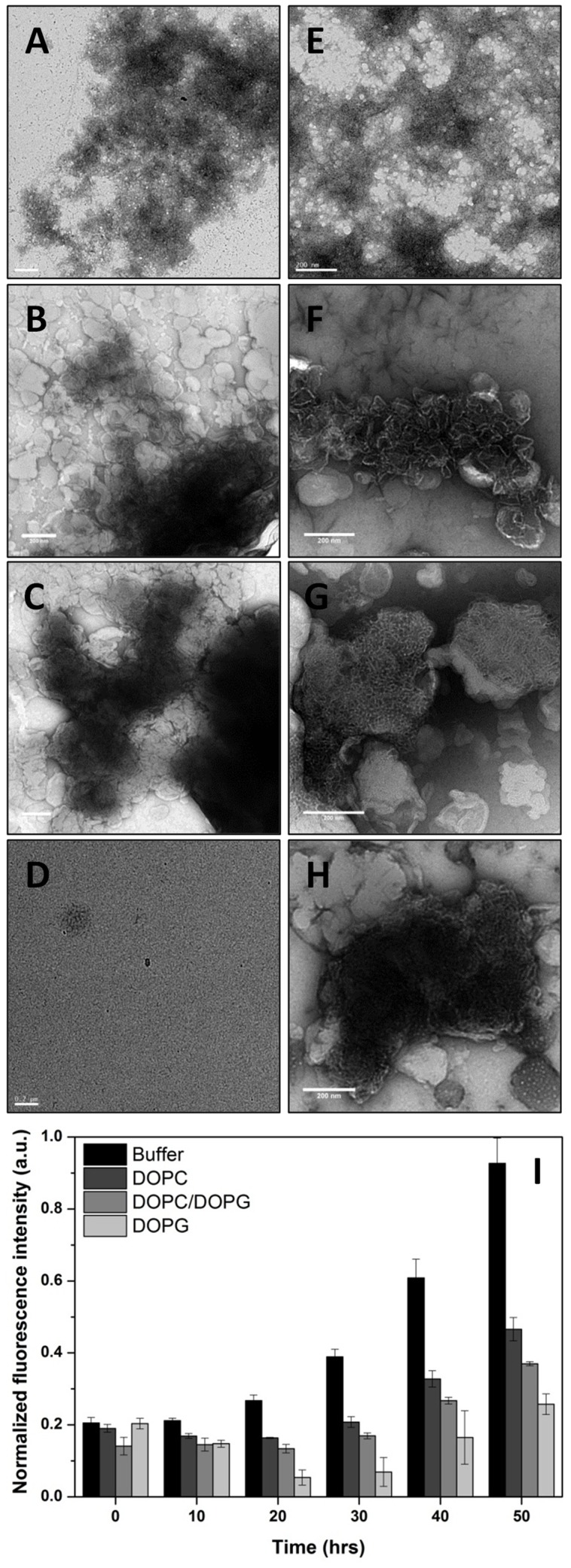
Negatively stained electron microscopy images of Drs S9 at 100 µM in solution (A and E), in the presence of DOPC (B and F), of DOPC/DOPG 7∶3 (C and G) and of DOPG (D and H) (scale bars 200 nm) after incubation for 1 day (A-D), 8 days (E-G) and 17 days (H). (I) Drs S9 fibril formation determined by ThT-fluorescence after incubation of 0, 10, 20, 30, 40 and 50 hours in solution (black), in DOPC LUVs (dark grey), in DOPC/DOPG LUVs (grey) and in DOPG LUVs (light grey).

Drs S9 has a propensity to form amyloid-like fibrils in solution at high concentration [14], although the aggregation process is found to be slow compared to amyloid peptides, typically requiring several hours at 500 µM [14]. Consistent with this, we observed the formation of fibrils for Drs S9 in solution at 100 µM after 1 day of incubation (Figures 3A-E). Interestingly, for the same peptide concentration approximately 1 to 8 days were required to obtain fibrils in the presence of DOPC and DOPC/DOPG vesicles and more than 15 days in the presence of DOPG vesicles (Figures 3B-H). In solution, the fibrils showed the typical morphology of amyloid fibrils with diameters between 10 and 15 nm, consistent with at least two filaments coiled around each other. In contrast, the population of Drs S9 fibrillar assemblies in vesicles consists of short, noodle-like fibrils that form thin but compact filamentous structures. The noodle-like fibrils seem even more packed in the presence of anionic lipid (Figures 3G-H).

Then, we followed the kinetics of fibril formation of Drs S9 in solution and in the presence of vesicles in more details by measuring the fluorescence intensity increase upon binding of the amyloid specific dye Thioflavin T (ThT), which is a commonly used method to detect amyloid fibrils [30]. ThT changes its fluorescence characteristics remarkably upon interaction with amyloids. The kinetics of fibril formation were followed at peptide concentrations of 100 µM at 25°C. Consistent with the EM results, Figure 3I shows that at 100 µM Drs S9 is able to form fibrils after a few days of incubation in solution, and that less fibril formation takes place in the presence of DOPC and in the presence of DOPC/DOPG, while Drs S9 hardly forms fibrils in the presence of DOPG. The overall observation that in the presence of lipids less fibrils are formed is consistent with the recent notion, that highly fibrillogenic peptides form fibrils more rapidly in the presence of lipid vesicles than in their absence, while the opposite is observed for peptides of low fibrillogenicity, where fibril formation is slower in the presence of lipid vesicles [31], [32].

### Incorporation method promotes α-helical conformation for Drs S9

**Figure 4:**
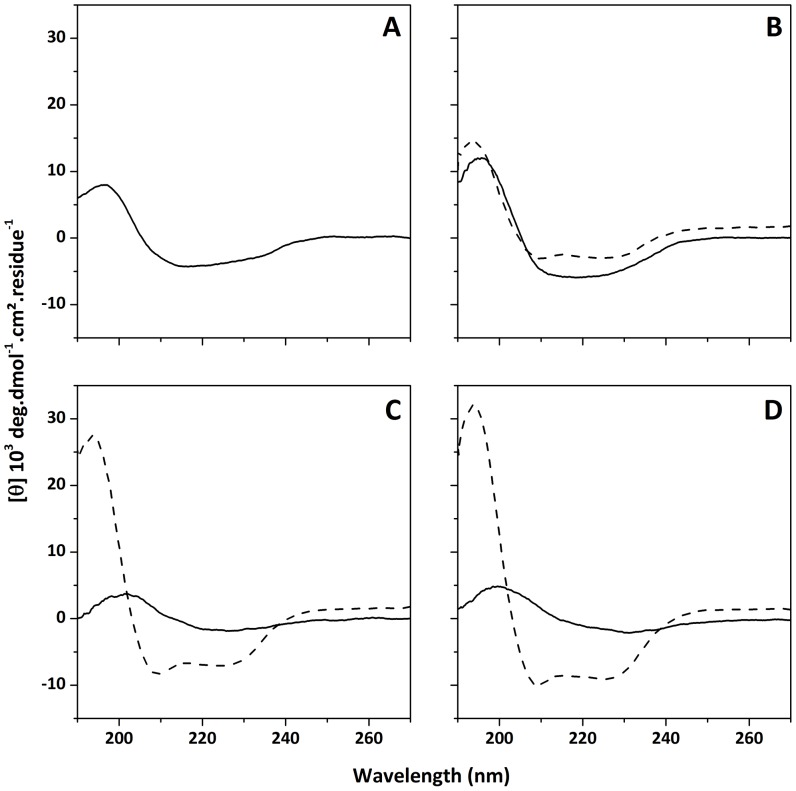
CD spectra (190–270 nm) of Drs S9 (25 µM) in (A) phosphate buffer at pH 7.4, (B) DOPC LUVs, (C) DOPC/DOPG 7∶3 LUVs, and (D) DOPG LUVS. Peptide was either added (solid line) or incorporated (dashed line) to vesicles. Peptide/lipid ratio was 1∶20.

Our data demonstrate that the membrane leakage and insertion is weaker in DOPG than in DOPC/DOPG and DOPC vesicles. A possible explanation is that this observed difference in Drs S9-membrane damage could be related to differences in aggregational behavior, which in turn could be related to differences in conformational behavior of the peptide. To study the secondary structure of Drs S9 in the absence and presence of membranes, we performed CD spectroscopy. For these experiments phosphate buffer without sodium chloride was used in order to increase the signal-to-noise ratio, keeping in mind that the absence of salt may affect peptide-membrane interactions. Drs S9 freshly dissolved in 10 mM phosphate buffer at 25 µM displays a peak with negative ellipticity at approximately 220 nm and a peak with positive ellipticity at 195 nm, that are characteristic of β-sheet structures (Figure 4A). Deconvolution of this spectrum allows to estimate the proportion of secondary structures as 9% α-helix, 41% β-sheet and 50% random coil. Extending the incubation time caused the CD signal to decrease (data not shown) proving that Drs S9 forms insoluble aggregated species that are not visible by CD spectroscopy.

When Drs S9 is added to vesicles (DOPC, DOPG or DOPC/DOPG), the CD signal is quite similar (Figures 4B-D, solid line) to the one obtained in phosphate buffer, indicating that the peptide also adopts β-sheet conformations in the presence of lipids. In the presence of DOPC, the intensity of the CD signal seems more dominant, suggesting a higher percentage of β-sheet, that is possibly related to its higher tendency to form fibrils. This is in agreement with our TEM and ThT experiments, which showed more amyloid-like fibrils in the presence of DOPC than in the presence of DOPC/DOPG or DOPG. After a few hours of incubation, the CD signal decreases which can be ascribed to Drs S9 aggregation (data not shown). These results are in agreement with SDS-Tris-Tricine-PAGE experiments which show the presence of small oligomeric forms for the peptide in solution and when added to lipid vesicles suggesting that Drs S9 self-associates [14].

To further study the importance of conformational behavior and amyloidogenicity for Drs S9/lipid interactions, we also used an "incorporation" protocol, in which Drs S9 was added to the phospholipids prior to the formation of the vesicles by co-dissolving peptide and lipids in organic solvent and drying them into a film that was subsequently hydrated to form the vesicles. This incorporation method is expected to favor peptide/lipid interactions over peptide/peptide interactions, thus reducing characteristic formation of aggregates in aqueous solution. CD spectra of Drs S9 incorporated into vesicles according to this protocol (Figures 4B–D, dashed line) display negative ellipticity at 208 and 225 nm, characteristic of α-helical structure. Deconvolution of the spectra allows the estimation of 40% in β-sheet in all the lipid vesicles and 16% α-helical structure in PC, 37% in DOPC/DOPG and 44% in DOPG, indicating that the α-helical content increases with DOPG concentration while the β-sheet content remains constant. These results provide evidence that PG-containing membranes promote an α-helical conformation for membrane-bound Drs S9.

### Interaction of Drs S9 with membrane determined by Differential Scanning Calorimetry

**Figure 5:**
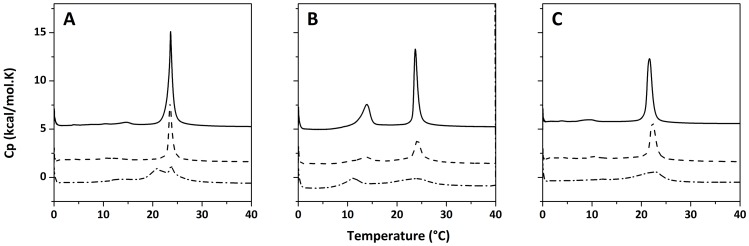
DSC thermograms illustrating the effect of the addition (dashed line) or the incorporation (dashed dotted line) of Drs S9 to (A) DMPC MLVs, (B) DMPC/DMPG 7∶3 MLVs, and (C) DMPG MLVs. Solid lines correspond to pure lipids.

Next, to gain insight into possible molecular mechanisms of leakage and to look more closely at the effects of the peptide on the organization of the lipids, the interaction of Drs S9 with membranes constituted of DMPC, DMPG or DMPC/DMPG phospholipids was characterized by differential scanning calorimetry. The phase behavior of lipid membranes is highly sensitive to the presence of external compounds, such as peptides [33]–[35]. Monitoring changes in thermodynamic parameters such as melting temperature T_m_ and enthalpy ΔH can provide information on the nature of the interactions between the peptide and the membrane. DSC thermograms illustrating the effect of Drs S9 on the thermotropic behavior of DMPC, DMPC/DMPG and DMPG MLVs are presented in Figure 5. Pure lipid scans (Figures 5A-C solid) present two distinct endothermic transitions: a more energetic event around 23°C corresponding to the chain melting transition (P_β’_ rippled gel phase to L_α_ fluid lamellar phase) and a less energetic event corresponding to the pre-transition (L_β’_ lamellar gel phase to P_β’_ rippled gel phase) near 13°C. The main transition peak is tall and narrow, indicating a large enthalpy and a highly cooperative transition. In contrast, the pre-transition, corresponding to the formation of rippled phase and the untilting of the lipid acyl chains, has a lower enthalpy and is less cooperative, with a smaller and broader peak. The addition of Drs S9 from aqueous solution to DMPC, DMPC/DMPG and DMPG (Figures 5A-C dash) leads to a temperature decrease of both the pre-transitions (which completely disappear in the case of DMPC and DMPG) and main transitions, indicating that the peptide interacts with lipid headgroups and slightly perturbs the acyl chains. Importantly, in DMPC the transition peak seems to remain sharpest, possibly indicative of more peptide aggregation and therefore less efficient membrane interaction. Pre-incorporation of the peptide to the vesicles via cosolubilization (Figures 5A-C dash dot) leads to significantly larger changes in the thermotropic behavior of the lipid bilayer. In the case of DMPC, incorporation of Drs S9 results in the appearance of a second transition at about 21°C that coexists with the main transition at 23°C. This suggests that two phases coexist, one corresponding to lipids in a peptide-poor region and another corresponding to lipids in a peptide-rich region, again indicative of peptide aggregation. In case of DMPC/DMPG and DMPG vesicles, the enthalpy and the cooperativity of the main transition are strongly decreased. Most likely in these cases Drs S9 localizes near the interface, where electrostatic interactions of the peptide with the anionic lipids may lead to a perturbation of the lipid headgroup area and hence to a disturbance of the cooperative behavior of the lipids. Furthermore, also with this protocol, the pre-transition disappears in the case of DMPC and DMPG, and is strongly decreased for DMPC/DMPG. Overall, the results indicate that Drs S9 inserts into lipid bilayers with differences between DMPC, DMPC/DMPG and DMPG, which are consistent with a higher tendency of the peptide to aggregate in the zwitterionic PC bilayers.

### Proton-decoupled ^31^P and ^2^H solid-state NMR spectra to monitor lipid macroscopic phase properties, structure and dynamics

**Figure 6:**
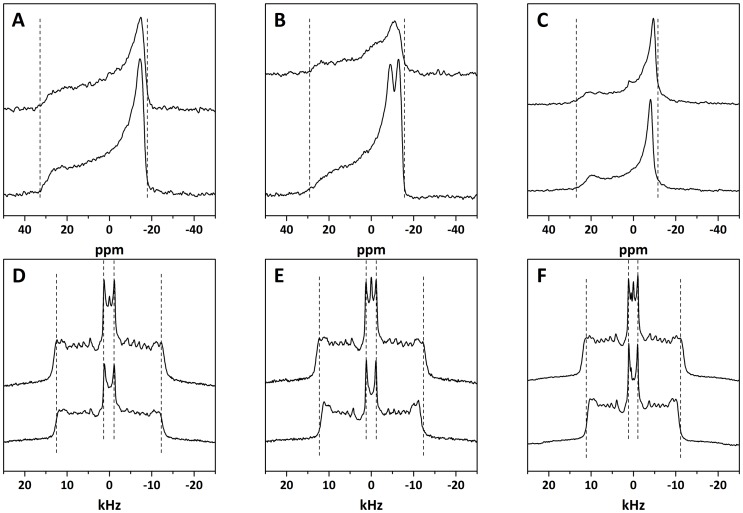
^31^P-NMR spectra of multilamellar vesicles composed of (A) POPC-d_31_, (B) POPC-d_31_/POPG 7∶3 and (C) POPG-d_31_. ^2^H NMR spectra of multilamellar vesicles composed of (D) POPC-d_31_, (E) POPC-d_31_/POPG 7∶3 and (F) POPG-d_31_. Experiments were performed in the absence (top) and in the presence (bottom) of Drs S9. Vertical dashed lines are eye guides for lipid ordering at plateau positions.

**Figure 7:**
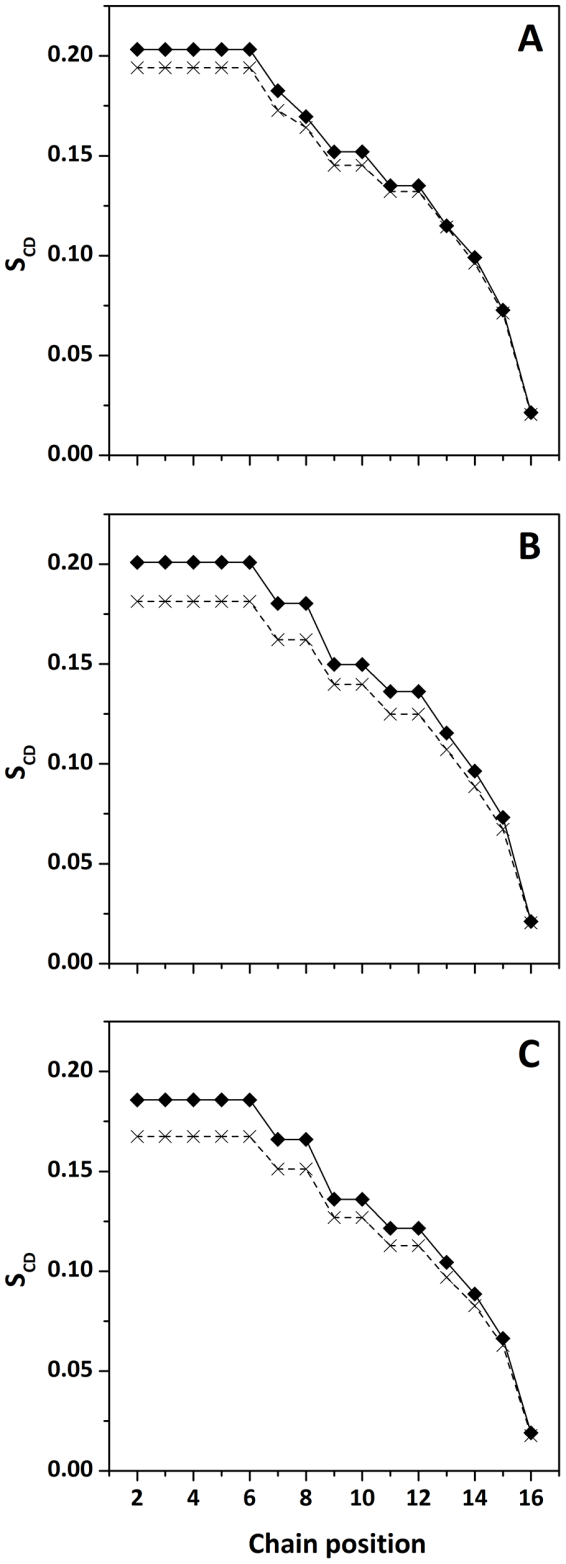
Order parameter profile calculated for de-Paked spectra of (A) POPC-d_31_, (B) POPC-d_31_/POPG 7∶3, and (C) POPG-d_31_, in the absence (diamond) and in the presence (cross) of Drs S9.

To obtain more details about the mode of membrane insertion of Drs S9 and the peptide/lipid interactions involved, we determined the influence of preincorporation of Drs S9 on lipid order by ^31^P-NMR and ^2^H-NMR. The peptide was incorporated into multilamellar vesicles of perdeuterated POPC-^2^H_31_, POPC-^2^H_31_/POPG and POPG-^2^H_31_ at a 1∶20 peptide:lipid molar ratio. ^31^P-NMR enables to probe Drs S9-membrane interactions at the headgroup level. Figure 6 (A-C) shows classical axially symmetric spectra of POPC-d_31_, POPC-d_31_/POPG and POPG-d_31_ bilayers, in the absence (top) and presence (bottom) of peptide. Spectra of pure lipids exhibit a typical pattern of lipid bilayers in a fluid lamellar phase at 25°C. Spectra of POPC-d_31_/POPG (Figure 6B) are the superposition of two subspectra originating from the phosphorous nuclei of POPC and POPG. Because of the different headgroup structures of the two lipids, each subspectrum shows a slightly different residual chemical shift anisotropy (CSA) [36], [37]. POPC typically has a CSA of ∼44 ppm and POPG of ∼32 ppm. The mixture contains a cumulative CSA of POPC and POPG. The addition of peptide (bottom) does not lead to major macroscopic changes in the shape of the spectra. In particular, there is no apparition of an isotropic peak, indicating that no small vesicles are formed. However, addition of the Drs S9 to each lipid system promoted a perturbation to the phospholipid head group region, with a 2 to 10% decrease in the CSA. Drs S9 shows evidence of significant membrane interaction, which leads to an increase in membrane fluidity. To obtain more information on the lipid order induced by Drs S9, we used ^2^H-NMR spectroscopy. Figure 6 (D-F) presents the ^2^H-NMR spectra of POPC-d_31_, POPC-d_31_/POPG and POPG-d_31_ bilayers, in the absence (top) and in the presence (bottom) of peptide at 25°C. In the case of pure lipids, we observe the typical spectra with many resolved splittings that are characteristic of lamellar fluid phases. The smallest splitting is about 3 kHz and corresponds to the mobile methyl groups at the end of the acyl chains, in the core of the bilayer. The largest one, corresponding to the CD_2_ groups closest to the glycerol backbone, is about 23–24 kHz, and is giving information about lipid order near interface. The addition of Drs S9 (bottom) does not lead to the formation of an isotropic peak, thus no small objects that would undergo fast tumbling are formed, in agreement with the ^31^P-NMR experiments. However, we observe a reduction of some of the ^2^H quadrupolar splittings. The more affected positions are those closest to the glycerol backbone, indicating that the peptide does not seem to insert deeply into the membrane. In the presence of Drs S9, the largest splitting decreases from 24.7 kHz to 23.5 kHz for POPC-d_31_, 24.5 kHz to 22.3 kHz for POPC-d_31_/POPG and 22.5 kHz to 20.3 kHz for POPG-d_31_. Thus, the perturbation near the polar headgroup seems to increase in the presence of POPG. Figure 7 shows the order parameter (S_CD_) for each carbon position in the lipid chain in the absence (diamonds) and in the presence (crosses) of peptide. The POPC–d_31_ chain order is lower (Figure 7A) in the presence of Drs S9, especially for the “plateau” positions (carbons 2 to 6). The effect is less marked until carbon 12, and is insignificant beyond. In POPC-d_31_/POPG and POPG-d_31_ liposomes (Figures 7B and 7C respectively), the order parameter of lipid chain is more reduced by Drs S9, affecting almost all carbon positions. The smaller effect on PC membranes would be consistent with a higher tendency of Drs S9 to aggregate in PC, and also indicates that the induced lipid disorder propagates more deeply in PG-containing membranes than in PC. In addition, the stronger electrostatic interactions of the peptide in case of PG containing membranes may lead to a larger perturbation of the lipid headgroup area, which would be consistent with the DSC results. In all lipid systems, the more affected positions are those corresponding to the CD_2_ groups closest to the glycerol backbone suggesting a shallow insertion of Drs S9 into the membrane. Overall, NMR experiments indicate that the presence of Drs S9 leads to a slight increase in membrane fluidity.

## Discussion

In the present study we have investigated and compared the mechanism of Drs S9-induced membrane disruption in relation with Drs S9-structure in lipid membrane models of different compositions. We have made the following key observations: i) Drs S9 induces more membrane leakage in PC than in PC/PG and PG vesicles; ii) Drs S9 is more fibrillogenic in PC than in PC/PG and PG; iii) smaller membrane perturbations are observed in the presence of PC than PC/PG and PG. These results are further discussed below.

We used negatively charged vesicles composed of PC/PG lipids to assess the ability of the peptides to permeabilize bacterial inner membrane, 100% of zwitterionic lipid and 100% of anionic lipid to provide information which helps to understand peptide/lipid interactions and to determine the role of electrostatic interactions. Because Drs S9 possesses five basic and one acidic residues at pH 7.4, leading to a cationic +4 global charge, a preferred interaction with the anionic lipid is probable. Unexpectedly, our results show that Drs S9 induces more leakage and inserts better in the presence of zwitterionic membrane (DOPC) than in the presence of negatively charged lipid (DOPC/DOPG and DOPG). This result is rather surprisingly, but a recent study showed that the highly cationic antimicrobial peptide MSI-78 (+9 global charge), also induces a very low leakage in pure PG vesicles [7]. The low leakage efficiency observed from pure PG vesicles could be due to strong electrostatic interactions between cationic residues of Drs S9 and negatively charged headgroups of PG that may anchor the peptide on the membrane surface, restricting its penetration into the bilayer core and thus its lytic activity, as it was proposed for other cationic antimicrobial peptides [7], [38]. This is consistent with the monolayer experiments showing that Drs S9 inserts less efficiently in PG membranes. Thus, it seems reasonable to assume that in the presence of 100% PG, Drs S9 somehow is anchored to the membrane surface. Thus, the electrostatic interactions with anionic lipids cannot be a determining factor to account for Drs S9 selectivity toward microorganisms membranes, which is unusual for a cationic peptide. Instead, our results show that in the presence of zwitterionic lipid, Drs S9 seems to induce membrane damage that is related to formation of small aggregates rather than fibril formation, as explained in the next paragraph.

The EM, ThT-fluorescence and CD studies of Drs S9 reveal several interesting features. The EM results indicate that the aggregates formed in solution by Drs S9 are analogous to disease-related amyloid fibrils [39]. Indeed, the Drs S9 fibrils exhibit the typical morphology of long and twisted amyloid fibrils with widths between 5 and 15 nm. However, unlike the amyloid peptides which assemble into amyloid fibrils in a few hours at 5 µM, the kinetics of Drs S9 fibril formation is very slow; several hours of incubation are required to observe the amyloid-like fibrils at 100 µM concentration. It is known that membranes containing anionic lipids increase the formation of amyloid fibril formation [40]. Our EM experiments in the presence of zwitterionic and anionic membranes did not show any evidence of amyloid-like fibril formation for Drs S9 after few days of incubation, but did show short and dense noodle-like fibrils. Noteworthy in the presence of 100% of anionic lipid, the noodle-like fibrils were more compact and were observed only after about 15 days. These results are consistent with the ThT-fluorescence and CD data in the presence of membranes that show small increases in ThT signal after few hours of incubation and β-sheet structure for Drs S9. Together with the EM data, these results indicate the following order of fibrillogenicity for Drs S9: DOPC > DOPC/DOPG > DOPG. Our data provide evidence that, unlike several antimicrobial peptides that form amyloid-like fibrils only in the presence of phospholipids [41]–[43], Drs S9 forms amyloid-like fibrils in both an aqueous and lipidic environment. Furthermore, the results demonstrate that Drs S9 amyloid-like aggregation on the membrane surface is not required for membrane permeabilization. Indeed, the membrane leakage starts after a few minutes with a peptide concentration of 1 µM, whereas the fibrils are formed after a few days at 100 µM. We can conclude that Drs S9 aggregation at the membrane surface is not essential for its cytotoxicity, unlike amyloid peptides for which it was shown that the growth of amyloid fibrils at the membrane surface causes membrane damage [44]–[46]. Nevertheless, the data suggest a relationship between aggregation and membrane damage, suggesting that membrane damage may be induced by small aggregates of peptide in β-sheet conformation, possibly precursors in the process of fibril formation.

Because many antimicrobial peptides affect the physicochemical properties of lipid membranes, we studied the lipid-peptide interactions by using DSC, ^31^P- and ^2^H-NMR methods. The addition of Drs S9 had little effect on the thermodynamics of the gel to liquid-crystalline phase transition. This indicates that the lipid-lipid interactions stabilizing the membrane are largely intact. It was shown that the aggregation of a peptide decreases the perturbation on the membrane by reducing the surface area of the membrane in contact with the peptide [47]. Self-association of the peptide lowers the number of lipid molecules in contact with the peptide and is therefore expected to decrease the influence of the peptide on the physical properties of the membrane. For that reason, we also studied the peptide-membrane interactions using the incorporation method, where the peptide is added prior to the formation of the vesicles. In this case, the incorporation of Drs S9 has a larger effect on the thermodynamics of the gel to liquid-crystalline phase transition suggesting that the peptide interacts strongly with the acyl chains of the lipids. In addition, the main phase transition of the lipid system is broadened, indicating a loss of lipid cooperativity. Our data show a quite similar enthalpy for the membrane in the absence and in the presence of Drs S9 indicating that the peptide does not perturb the membrane cohesion by changing the size of the vesicles as occurs with membrane fusion, micelle formation or breaking down the membrane [48]. These results are in agreement with the ^31^P-NMR results which show a conserved lamellar line shape and no dominant isotropic line. There is also no dominant isotropic line in deuterium spectra, clearly indicating that membrane integrity is maintained upon interaction with Drs S9. Incorporation of Drs S9 decreases the order parameter in all the lipid systems. The extent of decrease in the order parameter is higher for the CD_2_ groups closer to the head group region and decreases along the acyl chains towards a minimum for the other end of the chain. Thus, the peptide binding increases the disorder in the hydrophobic region of PC, PG and PC/PG bilayers with a maximal disorder closer to the glycerol backbone of the lipid. Negligible changes are observed in the order parameters of CD_2_ groups near the lower end of the lipid. Possibly, the hydrophobic core near the terminal methyl group of the bilayer is highly disordered even in the absence of the peptide and therefore, the peptide-induced disorder appears to be negligible. It is noteworthy that the membrane-disorder induced by Drs S9 is of the same order compared to that of other antimicrobial peptides [49]–[56]. The calcein experiments show a membrane leakage in membranes without and with limited content of anionic lipid (PC, PC/PG) suggesting that the leakage could be induced either by perturbation of lipid packing or by formation of peptides pores. Nevertheless, our DSC and solid-state NMR data do not show any huge perturbation of membrane cohesion, clearly indicating that membrane integrity is largely maintained upon interaction with Drs S9. SDS-Tris-Tricine-PAGE experiments confirm the presence of oligomeric forms of Drs S9 (data not shown). Our data suggest that the peptide binds to the membrane in an aggregated state. One possible hypothesis is that the peptide binds to the membrane with a transmembrane insertion and may induce a transient pore. Indeed, the results presented in this paper show that such a mode of pore formation may occur in membranes containing PG and PC that mimic the lipid composition of *E.coli*. However, it is unlikely that such pores are triggered by the presence of anionic lipids, as proposed for many other cationic antibiotics, since pores are not formed in pure anionic lipid bilayers. Therefore, we propose that other, yet unknown, factors must be involved to account for the antibiotic activity of Drs S9.
